# Supplementary material for: Deletion of wfs1 Impairs Oligodendrocyte Precursor Cells Dorsal Distribution and Myelination Through the wfs1-hmgcs1 Axis in Zebrafish
Source: Biology (Basel). 2026 Aug 16;15(16):1402. doi: 10.3390/biology15161402 (PMC13510151; doi:10.3390/biology15161402)
Supplement: Supplementary file 1 [file biology-15-01402-s001.zip › File S1-Transcriptome sequencing data and differentially expressed genes (DEGs) in wfs1-- zebrafish at 5 dpf/2.KEGG/wfs1KOvsWT/DOWN/src/dre04514.html]

dre04514


- dre:557935

- Down regulated genes

557935(-1.87319484154862)

- dre:797491

- Down regulated genes

797491\_1(-1.09418588609297)

- dre:797651

- Down regulated genes

797651(-1.27962292749959)

- dre:797651

- Down regulated genes

797651(-1.27962292749959)

- dre:557935

- Down regulated genes

557935(-1.87319484154862)

- dre:797651

- Down regulated genes

797651(-1.27962292749959)

- dre:797651

- Down regulated genes

797651(-1.27962292749959)

- dre:557935

- Down regulated genes

557935(-1.87319484154862)

- dre:557935

- Down regulated genes

557935(-1.87319484154862)

- dre:557935

- Down regulated genes

557935(-1.87319484154862)

- dre:557935

- Down regulated genes

557935(-1.87319484154862)

- dre:569084

- Down regulated genes

569084\_1(-2.40277205964249)
- dre:81585

- Down regulated genes

81585(-1.74514319517014)
- dre:60635

- Down regulated genes

60635\_1(-1.07799587140666)
- dre:110438642

- Down regulated genes

110438642(-3.83724028917273)

- dre:569084

- Down regulated genes

569084\_1(-2.40277205964249)
- dre:81585

- Down regulated genes

81585(-1.74514319517014)
- dre:60635

- Down regulated genes

60635\_1(-1.07799587140666)
- dre:110438642

- Down regulated genes

110438642(-3.83724028917273)

- dre:569084

- Down regulated genes

569084\_1(-2.40277205964249)
- dre:81585

- Down regulated genes

81585(-1.74514319517014)
- dre:60635

- Down regulated genes

60635\_1(-1.07799587140666)
- dre:110438642

- Down regulated genes

110438642(-3.83724028917273)

- dre:797651

- Down regulated genes

797651(-1.27962292749959)

- dre:557935

- Down regulated genes

557935(-1.87319484154862)

- dre:569084

- Down regulated genes

569084\_1(-2.40277205964249)
- dre:81585

- Down regulated genes

81585(-1.74514319517014)
- dre:60635

- Down regulated genes

60635\_1(-1.07799587140666)
- dre:110438642

- Down regulated genes

110438642(-3.83724028917273)

- dre:101886406

- Down regulated genes

101886406(-1.56097230660967)

Close
